# Assessing Social Identity in Autistic Individuals: Evaluating A Self-Report Questionnaire in the Netherlands

**DOI:** 10.1177/13623613261431269

**Published:** 2026-04-03

**Authors:** Lisa JG Krijnen, Ralph CA Rippe, Sander Begeer, Rachel D Plak

**Affiliations:** 1 Leiden University, The Netherlands; 2 Vrije Universiteit Amsterdam, The Netherlands; 3 Amsterdam Public Health Research Institute, The Netherlands

**Keywords:** autism, autistic identity, mental health, psychometrics, questionnaire, social identity

## Abstract

People with autism often face mental health difficulties at rates far exceeding those of the general population. How autistic individuals relate to their autism classification and the autistic community, also known as *social identity*, may form a protective factor for mental health. However, validated tools to assess social identity in autistic populations are lacking. This study aims to evaluate the Dutch version of the 14-item Social Identity in Autism Questionnaire (SIAQ) and examine associations between social identity and demographic, autism-related, and mental health variables. A total of 1443 autistic individuals from the Netherlands (mean age = 47 years, 54% women, 98% Dutch) completed the SIAQ and measures assessing demographics, autism characteristics, and mental health. Factor analyses revealed a four-factor structure: *solidarity* (three items, feelings of connection to people with autism), *satisfaction* (four items, positive feelings about being autistic), *centrality* (three items, the importance of autism to one’s sense of self), and *self-definition* (four items, perceived similarity to other autistic people and within the autistic community). Internal consistency was acceptable to excellent. Measurement invariance (scalar level) was found across age, gender, education level, ethnicity, and autism traits. Furthermore, the four factors of social identity were differentially related to age, gender, language preference, time since diagnosis, and autism traits. Higher satisfaction and lower centrality were related to better mental health. To conclude, the SIAQ forms a robust tool to assess social identity in autistic individuals in the Netherlands.

**Lay Abstract:**

People with autism experience mental health challenges much more often than people in the general population. Understanding how autistic people relate to their autism and the autistic community – called autistic *social identity* – may form an important factor for mental health. However, the lack of reliable tools to measure social identity in autistic people led to this study evaluating the Dutch version of the Social Identity in Autism Questionnaire (SIAQ). Associations between social identity and demographics, autism traits, and mental health were studied. Autistic individuals from the Netherlands (*n* = 1443, average age = 47 years; 54% women; 98% Dutch) completed the SIAQ. The results showed that the questionnaire captures four key aspects of social identity: solidarity (feeling connected to other autistic people), satisfaction (positive feelings about being autistic), centrality (how central autism is to one’s identity), and self-definition (seeing oneself as similar to other autistic people and perceiving the autistic community as relatively similar). The questionnaire was reliable as well as suitable to use across diverse groups, including variations in age, gender, education level, ethnicity, and autism traits. Several aspects of social identity were related to gender, age, language preference, time since diagnosis, and autism traits. Importantly, higher satisfaction and lower centrality were associated with better mental health. These findings suggest that in the Netherlands, the SIAQ is a useful tool for understanding how autistic people relate to their autism and the autistic community, and how this relates to wellbeing.

## Introduction

People with autism^1^ often face mental health difficulties at rates far exceeding those of the general population. For example, 27% of the people with autism currently experience a depressive disorder, and 23% an anxiety disorder, which is substantially higher than the 1%–12% found in the general population (Hollocks et al., 2019; Kessler et al., 2012). Suicide rates are alarmingly elevated, with a two- to ten-fold higher risk of death by suicide among autistic people than among nonautistic peers (Bentum et al., 2024; Brown et al., 2024). Moreover, autistic individuals ranked mental health as the top research priority (Cage et al., 2024; Onderzoeksagenda Autisme, 2018). It is therefore urgently needed to respond to their priorities by studying factors related to wellbeing.

### Societal Perceptions of Autism

According to the *Diagnostic and Statistical Manual of Mental Disorders, Fifth Edition* (*DSM*-5; American Psychiatric Association, 2013), autism spectrum disorder, hereafter autism, is a neurodevelopmental *disorder* characterized by *deficits* in social functioning and interaction, in addition to restrictive and repetitive interests and behaviors. Describing autism as a *disorder*, a term that inherently carries a connotation of abnormality or dysfunction, frames autism as something undesirable. Although such descriptions are designed for diagnostic clarity, they can also contribute to a deficit-oriented view of autism. Media representations may contribute to deficit-based perceptions of autism, with movies and television showing negative and stereotypical portrayals of autism, potentially influencing societal views (Mittmann et al., 2024). When these deficit-oriented views are echoed in society, they can affect how autistic individuals view themselves (Botha & Frost, 2020). Over the past decade, a shift toward the strength-based approach has gained attention, highlighting unique strengths of people with autism (Murthi et al., 2023). This paradigm shift from ‘deficits’ to strengths may help foster a more positive self-image.

### Self-perception and Autism

Developing a positive self-image is especially relevant during adolescence, when identity development plays a central role and young people question who they are, how they differ from others, and which groups they belong to (Van Doeselaar et al., 2018). Receiving an autism classification during this developmental phase can be challenging, as young people have to integrate autism into their self-concept (Davies et al., 2024). Some adolescents embrace their autistic identity, while others desire to be ‘normal’ (Davies et al., 2024; Humphrey & Lewis, 2008; Mogensen & Mason, 2015).

However, many autistic individuals receive a formal classification much later in life, particularly women, ethnic minorities, and those without intellectual disabilities (Huang et al., 2021; Wiggins et al., 2020). For them, the diagnostic process involves reconstructing their life narrative and understanding past experiences through a new lens, which can evoke complex emotions ranging from relief and validation to confusion and grief (Leedham et al., 2020; Lewis, 2016). These differences in timing of diagnosis and lived experiences show that autistic people can relate to their autism in diverse ways. This diversity is important to consider when examining how individuals’ perspectives on their autism are associated with identity and wellbeing.

### Social Identity

Understanding how autistic individuals relate to their autism and to the autistic community, a concept known as autistic *social identity*, may offer a valuable framework for understanding wellbeing in autistic individuals (Davies et al., 2024; Maitland et al., 2021). According to the Social Identity Theory (Tajfel & Turner, 1979), one’s social identity, which is derived from the perceived membership of social groups (e.g. a student, a soccer player, a European citizen, etc.), provides a sense of belonging, connection, and support. These positive outcomes are likely to enhance self-esteem and wellbeing (Greenaway et al., 2015; Jetten et al., 2017). However, when one’s social group is being stigmatized and discriminated, identifying with this group can lead to vulnerability and isolation (Kellezi & Reicher, 2012). Given that the autistic community can be considered a social group that one can identify with, it becomes relevant to understand how this relates to mental health.

### Assessing Social Identity

Although studies in different countries have investigated social identity in autism, reliably measuring this remains a key challenge. One promising tool has been developed by Leach et al. (2008), which can be adapted to different social identities or in-groups. This 14-item self-report questionnaire captures five subcomponents of social identity, grouped into two broader dimensions. The first dimension, *self-investment*, involves emotional and personal significance about group membership and consists of three subcomponents: solidarity (e.g. ‘I feel a bond with [in-group]’), satisfaction (e.g. ‘I am glad to be [in-group]’), and centrality (e.g. ‘I often think about the fact that I am [in-group]’). The second dimension, *self-definition*, reflects perceived similarity with(in) the group and includes two subcomponents: individual self-stereotyping (e.g. ‘I have a lot in common with the average [in-group]’) and in-group homogeneity (e.g. ‘[In-group] people have a lot in common with each other’). Leach et al. (2008) demonstrated high internal consistency across various identities. Cooper et al. (2017) adapted this questionnaire to assess autistic social identity, here referred to as the Social Identity in Autism Questionnaire (SIAQ), and has been used in several autism studies afterwards (e.g. Ferenc et al., 2023; Maitland et al., 2021).

Cooper et al. (2017) used the SIAQ to study social identity in an autistic sample in the United Kingdom. Their results suggested that a positive autistic social identity may form a protective mechanism for mental health, as they found that a stronger autistic identity was related with higher self-esteem and in turn to lower anxiety and depressive symptoms. In contrast, a study among Polish autistic participants found the opposite: Higher levels of autistic social identity was related with more psychological distress (Ferenc et al., 2023).

These different results may be due to how they measured social identity. Although they both used the SIAQ to assess social identity, they grouped the items in different ways into (sub)scales. Cooper and colleagues (2017) grouped all items into one scale, whereas Ferenc and colleagues (2023) created two subscales and omitted a few items for theoretical reasons. The different operationalization of social identity poses major challenges in interpreting and comparing their results. Given the limited research on how to assess social identity in autistic individuals, it is not yet clear which subscales best capture this construct.

In addition, cultural differences between countries can also explain different findings: How autism is portrayed and understood varies across countries (Kim, 2012). How people relate to their autism may therefore mean something else in different cultures, which has consequences for what the construct of social identity involves, how it should be assessed, and how it relates to mental health. One of the examples that illustrates cultural differences in social identity is language: countries differ in the extent to which autistic people prefer identity-first language (e.g. ‘autistic person’) versus person-first language (e.g. ‘person with autism’) (Buijsman et al., 2023; Taboas et al., 2023). Being aware of potential cultural differences is important when investigating how social identity should be measured.

### Study Aims

The first aim of the current study is to investigate whether the SIAQ is suitable to measure social identity among autistic individuals in the Netherlands. Using a large autistic sample, we will evaluate the SIAQ’s psychometric properties, starting with an exploration of its domain structure. This will clarify which aspects of social identity are measured and how subscales should be created when measuring autistic social identity in the Netherlands. The internal consistency will be assessed to determine unidimensionality of each subscale. Finally, measurement invariance will be evaluated to examine whether the SIAQ operates similarly across different subgroups (i.e. gender, age, education level, ethnicity, and autism traits).

The second aim of this study is to explore how social identity is associated with demographic (i.e. gender, ethnicity, education level, intelligence level, employment status, urbanicity), autism-related (i.e. autism traits, time since diagnosis, age of diagnosis, preference for identity-first or person-first language), and mental health variables (i.e. co-occurring conditions, anxiety, and depressive symptoms). This will provide insight into which characteristics are related with a stronger social identification with autism, and which aspects of social identity are related to mental health. Given the limited research within this topic, this study will be explorative in nature. It is important to note that in this study, we focus on the Dutch context, and although our findings may not be generalizable to other cultural settings, they offer an important contribution by examining the SIAQ in a Dutch-speaking sample and laying the groundwork for future cross-cultural investigations.

## Methods

### Participants

The sample consisted of 1443 participants (aged 16–84, mean age = 47.00, 54.30% women, 98% Dutch). Inclusion criteria were (1) age ≥16, (2) an official autism diagnosis according to the *Diagnostic and Statistical Manual of Mental Disorders, Fourth Edition* (*DSM*)-IV (American Psychiatric Association, 1994) or *DSM*-V (American Psychiatric Association, 2013) as established by a qualified professional independent of the current study, and (3) sufficient Dutch proficiency to complete the questionnaires. See Table 1 for the participants’ characteristics.

**Table 1.** Sample Characteristics (*n* = 1443).

Age^a^	
Mean (*SD*)	47.02 (14.19)
Range	16.72–83.90
Gender identity	
Man, *n* (%)	470 (32.60%)
Woman, *n* (%)	783 (54.30%)
Gender diverse, *n* (%)	190 (13.20%)
Education level^b^	
Low, *n* (%)	129 (8.94%)
Medium, *n* (%)	490 (33.96%)
High, *n* (%)	803 (55.56%)
*No response, n (%)*	*21 (1.46%)*
Parental education level^c^	
Low, *n* (%)	548 (38.00%)
Medium, *n* (%)	449 (31.10%)
High, *n* (%)	204 (14.10%)
*No response, n (%)*	242 (16.8%)
Intelligence level^d^	
Below average, *n* (%)	100 (6.9%)
Average, *n* (%)	285 (19.8%)
Above average, *n* (%)	1006 (69.7%)
*No response/don’t know, n (%)*	*52 (3.60%)*
Employment status^e^	
Employed, *n* (%)	695 (48.20%)
Unemployed, *n* (%)	737 (51.05%)
*No response, n (%)*	11 (0.76%)
Living area^f^	
North, *n* (%)	135 (9.40%)
East, *n* (%)	293 (20.30%)
South, *n* (%)	218 (15.10%)
West, *n* (%)	561 (38.90%)
*No response*	*236 (16.40%)*
Urbanicity	
Urban, *n* (%)	678 (47.00%)
In between urban and rural, *n* (%)	237 (16.40%)
Rural, *n* (%)	292 (20.20%)
*No response, n (%)*	*236 (16.40%)*
Ethnicity	
Dutch, *n* (%)	1172 (81.2%)
Other, *n* (%)	36 (2.5%)
*No response, n (%)*	*235 (16.30%)*
Age of autism diagnosis^g^	
Mean (*SD*)	36.81 (15.14)
Range	1–71.92
Time since autism diagnosis^h^	
Mean (*SD*)	10.02 (7.23)
Range	0.28–52.90
Autism-Spectrum Quotient-Short score^i^	
Mean (*SD*)	82.54 (10.74)
Range	31.00–112.00
Language preference^j^	
Identity-first, *n* (%)	137 (9.49%)
Person-first, *n* (%)	395 (27.37%)
Other, *n* (%)	41 (2.84%)
*No response, n (%)*	870 (60.29%)
Co-occurring conditions^k^	
Yes, *n* (%)	679 (47.05%)
No, *n* (%)	679 (47.05%)
*No response/don’t know, n (%)*	85 (5.89%)
Anxiety and depressive symptoms^l^	
Anxiety, mean (*SD*)	9.22 (4.44)
Depression, mean (*SD*)	7.08 (4.66)

a Five participants missing.

b Education level was divided into low/medium/high according to (Centraal Bureau voor de Statistiek, 2021). For participants aged ≤30 years enrolled in education, current enrollment used if higher than completed level, or if information regarding completed education was missing.

c Highest education of either parent presented.

d IQ based on test or self-estimate: above average >115, average 86–115, below average <86.

e Employed consisted of regular paid employment, self-employed, or entrepreneurship.

f Distribution of residents per area in the Netherlands: North: 9.9%; East: 21.10%; South: 21.10%; West: 48.0% (Centraal Bureau voor de Statistiek [CBS], 2024).

g Sixty-eight participants missing.

h Seventy participants missing.

i Autism indicative score >65 (Hoekstra et al., 2011). Eighty-nine participants scored below this cutoff score. Thirty-three participants missing.

j Identity-first: ‘Autists/Autistics’ or ‘Autistic people’. Person-first: ‘People with autism’ or ‘Someone with Asperger’s, PDD-NOS, or McDD’. Open answers were recoded into the existing categories.

k Co-occurring conditions included: neurodevelopmental or psychiatric conditions, neurocognitive variations, neurological condition, sensory impairments, physical/motor disabilities; genetic/chromosomal conditions, and other clinically significant conditions.

l The Hospital Anxiety and Depression Scale (HADS) was used. Scores 8–10: possible anxiety or depressive disorder. Scores ≥ 11 probable disorder (Zigmond & Snaith, 1983). Two participants missing.

### Procedure

Participants were derived from the Netherlands Autism Register (NAR; https://nar.vu.nl/). The NAR is a large, Dutch database containing information of autistic and nonautistic participants. The NAR collects data annually, by sending online questionnaires via e-mail. Before participants register at the NAR, they sign an informed consent.

NAR data collection has been approved by the Permanent Committee on Science and Ethics (VCWE) of the Vrije Universiteit Amsterdam (number: VCWE-2020-041R1). This study was preregistered at AsPredicted #208624, see https://aspredicted.org/w42b-8ddg.pdf. The code used for our analyses is available on Open Science Framework: https://osf.io/34rbp/overview?view_only=e5bff52f747d4b7aa67df3a18c5c0275.

### Participatory Methods

The NAR collaborates directly with autistic individuals and values the perspectives of the autism community. The NAR receives ongoing input through an advisory panel, consisting of 10–20 autistic people or parents of autistic children. The advisory panel provides input on research priorities, study design including clarity of questions, and potentially overlooked perspectives and biases. All annual questionnaires are reviewed by autistic individuals before distribution. Furthermore, the research team of the NAR also consists of autistic and nonautistic researchers, who provide feedback on new and ongoing studies of the NAR. For more information, see the protocol paper of the NAR (Jonkman et al., 2025).

### Materials

#### Social Identity in Autism Questionnaire

The SIAQ measures identification with autism, using 14 items with a 7-point Likert-type answer scale (1 = ‘*Strongly Disagree*’; 7 = ‘*Strongly Agree*’), with higher scores indicating stronger identification (Cooper et al., 2017; Leach et al., 2008). Cooper and colleagues (2017) adapted this questionnaire to autistic identity, based on the Multi-dimensional Scale of Social Identification (Leach et al., 2008). We translated Cooper and colleagues’ questionnaire into Dutch, thereby using both person-first and identity-first language to reflect differing language preferences in the Netherlands (Buijsman et al., 2023). The NAR includes autistic researchers as well as a panel of autistic people that routinely reviews questionnaires before being distributed to ensure clarity and appropriateness for the autistic community. Furthermore, a native English speaker fluent in Dutch was asked for feedback during the translational process.

Leach and colleagues (2008) investigated the factor structure of the Multi-dimensional Scale of Social Identification among various identities (i.e. Dutch identity, European identity, university identity). Using confirmatory factor analysis (CFA), Leach and colleagues (2008) identified a five-domain model structure, organized within two more general dimensions: self-investment and self-definition. Self-investment consisted of solidarity (Items 1–3, e.g. ‘I feel a bond with [in-group]’), satisfaction (Items 4–7, e.g. ‘I am glad to be [in-group]’), and centrality (Items 8–10, ‘I often think about the fact that I am [in-group]’). Self-definition consisted of individual self-stereotyping (Items 11–12, e.g. ‘I have a lot in common with the average [in-group]’) and in-group homogeneity (Items 13–14, e.g. ‘[In-group] people have a lot in common with each other’). Internal consistency was shown to be high (Leach et al., 2008).

#### Autism-Spectrum Quotient-Short (AQ-Short)

The AQ-Short assesses autistic traits, using 28 items answered on a 4-point Likert-type scale (1 = ‘*Definitely agree*’ to 4 = ‘*Definitely not agree*’) (Hoekstra et al., 2011). Higher scores reflect more autistic traits, with a score of >65 being indicative of autism (Hoekstra et al., 2011). High sensitivity and specificity have been reported (Hoekstra et al., 2011). In the current study, internal consistency was good (α = 0.83).

#### Hospital Anxiety and Depression Scale

The Dutch translation of the 14-item Hospital Anxiety and Depression Scale (HADS) was used for inquiring about anxiety (seven items, e.g. ‘I get sudden feelings of panic’) and depressive symptoms (seven items, e.g. ‘I feel as if I am slowed down’) over the past week, using a 4-point Likert-type scale (0 = ‘*Not at all*’ to 3 = ‘*Most of the time*’) (Spinhoven et al., 1997; Zigmond & Snaith, 1983). Higher scores indicate more symptoms. The HADS was shown to be reliable and valid in an autistic sample (Uljarević et al., 2018). In the current study, internal consistency was good (α = 0.86 for both scales).

### Statistical Analyses

R version 4.1.2 (through RStudio) and SPSS version 28 were used to analyze the data. The domain structure of the SIAQ was assessed to identify which subscales of the Dutch SIAQ should be distinguished. First, we carried out an exploratory factor analysis (EFA) to a random 60% participant subset to obtain a suggested domain structure. Second, we evaluated this specific suggested structure only for actual fit using CFA in the remaining 40% of the participants. EFA and CFA results were interpreted using standard criteria for sampling adequacy (EFA; Measure of Sampling Adequacy (MSA) > 0.50, Bartlett *p* < 0.05), factor retention (EFA; eigenvalues > 1.00, parallel analysis), and model fit (CFA; Chi^2^ test: nonsignificant result, Comparative Fit Index (CFI) > 0.95, Tucker–Lewis Index (TLI) > 0.95, Root Mean Square Error of Approximation (RMSEA) < 0.08, Standardized Root Mean Square Residual (SRMR) < 0.08) (Field et al., 2012; Hu & Bentler, 1999).

Internal consistency was assessed to quantify the degree to which items correlated with all other items of the same (sub)scale, where a higher correlation is indicative of measuring a unidimensional concept. We computed Cronbach’s Alpha for each subscale in both the EFA subsample and the CFA subsample for robustness. Values were interpreted as follows: >0.70 acceptable, >0.80 good, >0.90 excellent (Taber, 2018).

Measurement invariance was tested to assess to what degree the factor structure can be quantified the same way across different groups (i.e. gender, education level, ethnicity, age, and AQ score). We evaluated this using increasing restrictions on the confirmatory factor models in all groups under comparison, using the complete sample (i.e. EFA + CFA subsets together). Level 1 is configural invariance, forcing equal factor structure across groups, Level 2 is metric invariance, forcing also equal factor loadings across groups, and Level 3 is scalar invariance, forcing also equal intercepts across groups. If scalar invariance is reached, scores on the SIAQ scales can be meaningfully compared across groups. Configural invariance was evaluated using absolute fit criteria (CFI/TLI > 0.90, RMSEA/SRMR < 0.08), while metric and scalar invariance required both absolute fit criteria and at least two of three relative change criteria: ΔCFI ≤ 0.01, SRMR ≤ 0.01, ΔRMSEA ≤ 0.015 (Ubels & Schlander, 2023; van de Schoot et al., 2012).

Associations of SIAQ (sub)scales with demographic, autism-related, and mental health variables were evaluated using nonparametric methods to guard against violations of normality. Spearman rank-order correlations, which do not rely on variance and thus not on normality, were used for continuous and binary variables. Spearman’s correlations of <0.30 were considered small, 0.30–0.50 moderate, and >0.50 strong (Cohen, 1988). Kruskal-Wallis tests with Dunn’s post-hoc tests were conducted for categorical independent variables with at least three categories. Using *η*^2^ as the effect size, values for *η*^2^ < 0.06 were considered as small, 0.06–0.14 as medium, and >0.14 as strong (Cohen, 1988). Correction for multiple testing was applied for the main tests (i.e. Kruskal-Wallis and Spearman’s correlation) by adjusting the significance threshold: 5% was adjusted by the number of demographic, autism-related, and mental health variables tested (0.05/14 = 0.0036).

More technical details, decisions, and applied thresholds regarding each of the analyses above are provided in the Supplemental Materials.

## Results

### Domain Structure

#### Exploratory Factor Analysis

The random subsample of 60% of the data (*n* = 866) was suitable to perform an EFA, based on correlational inspection of the items, the determinant (0.0003), Bartlett’s test (<0.001), and the Kaiser-Meyer-Olkin test (overall MSA = 0.81, MSA for each item ≥0.75).

Based on the initial eigenvalues (four components with Sum of Squared (SS) loadings > 1.00: 4.86, 2.53, 1.58, 1.41) as well as the parallel analysis, a four-factor model was retained, explaining 64% of the variance. In Table 2, the factor loadings after oblique (oblimin) rotation (pattern matrix: factor loadings for each item on each factor), the SS loadings, and the variance explained per factor are shown. The interfactor correlations are presented in Supplemental Table 1.

**Table 2.** Factor Loadings (Pattern Matrix) of the Four-Factor Model of the EFA (*n* = 866) And The CFA (*n* = 577).

#	Item	Solidarity		Satisfaction		Centrality		Self-definition	
		EFA	CFA	EFA	CFA	EFA	CFA	EFA	CFA
1	I feel a bond with other people with autism	**0.83**	**0.83**	0.01		−0.01		0.02	
2	I feel solidarity with the autistic community	**0.75**	**0.81**	0.01		0.07		0.00	
3	I feel committed to people with autism	**0.97**	**0.91**	0.00		−0.02		0.00	
4	I am glad to be autistic	−0.01		**0.89**	**0.89**	−0.01		0.00	
5	I think that people with autism have a lot to be proud of	0.15		**0.57**	**0.59**	0.05		−0.06	
6	It is pleasant to be a person with autism	−0.03		**0.91**	**0.89**	−0.01		−0.01	
7	Being autistic gives me a good feeling	−0.02		**0.90**	**0.91**	0.02		0.04	
8	I often think about the fact that I am a person with autism	0.04		−0.20		**0.59**	**0.42**	0.03	
9	The fact that I am autistic is an important part of my identity	−0.01		0.01		**0.82**	**0.76**	0.00	
10	Being a person with autism is an important part of how I see myself	0.01		0.06		**0.85**	**0.89**	0.01	
11	I have a lot in common with the average autistic person	0.05		0.01		0.06		**0.78**	**0.82**
12	I am similar to the average person with autism	−0.05		−0.02		−0.01		**0.87**	**0.81**
13	People with autism have a lot in common with each other	0.13		0.03		−0.02		**0.56**	**0.67**
14	Autistic people are very similar to each other	−0.04		0.05		−0.05		**0.62**	**0.59**
Eigenvalues % of variance		2.30		2.83		1.78		2.11	
		16%		20%		13%		15%	

Bold values indicate factor loadings of >0.40. EFA = exploratory factor analysis; CFA = confirmatory factor analysis.

The four factors made theoretical sense and are in line with previous research (Leach et al., 2008; Maitland et al., 2021).

#### Confirmatory Factor Analysis

The CFA (*n* = 577) confirmed the four-factor model and showed acceptable fit: CFI = 0.93, TLI = 0.90, SRMR = 0.07, and RMSEA = 0.09. The Chi^2^ test was significant, χ²(71) = 398.79, *p* < 0.001. However, Chi^2^ tests are sensitive to sample size and frequently significant in large samples, even when other metrics indicate adequate fit (Brown, 2015). In Table 2, the factor loadings are presented, and in Supplemental Table 1, the interfactor correlations are shown.

### Internal Consistency

Results of both the EFA/CFA samples showed acceptable to good internal consistency: solidarity α = 0.89/0.88, satisfaction α = 0.90/0.89, centrality α = 0.79/0.72, self-definition α = 0.82/0.82.

### Measurement Invariance

According to the scaled fit indices as well as the standard fit indices (presented in Supplemental Tables 2 and 3), scalar invariance was found for all tested axes: gender, education level, ethnicity, age, and AQ score. The Chi^2^ test yielded significant results (all *p*’s < 0.001), but given that this test generally produces significant results in large sample sizes, this result is not considered as an indication for measurement variance.

### Associations With Variables

#### Descriptives SIAQ

The means and standard deviations of the SIAQ components are displayed in Table 3, and for each individual item in Supplemental Table 4.

**Table 3.** Mean Scores and Standard Deviations Per SIAQ Component.

Component	Mean (*SD*)
Solidarity	5.01 (1.25)
Satisfaction	3.62 (1.37)
Centrality	5.12 (1.22)
Self-definition	3.65 (1.09)

#### Associations With SIAQ Components

Kruskal-Wallis tests examined relationships between SIAQ components and nominal variables (see Table 4), revealing significant differences of a small effect for gender and language preference. Differences in satisfaction scores emerged between gender identity groups (*H* (2) = 11.81, *p* = 0.0027, *η*² = 0.007). Satisfaction scores were higher for man (*M* = 3.79, *SD* = 1.41) than for woman (*M* = 3.51, *SD* = 1.34), *Z* = 3.40, *p*_bonferroni_ = 0.002. Differences in centrality scores were observed for language preference (*H*(2) = 11.75, *p*_bonferroni_ = 0.0028, *η*² = 0.017). Centrality scores were higher for those who had a preference for identity-first language (*M* = 5.29, *SD* = 1.12) as opposed to those with a preference for person-first language (*M* = 4.91, *SD* = 1.25), *Z* = −3.16, *p*_bonferroni_ = 0.0047, and ‘other’ preferences (*M* = 4.63, *SD* = 1.47), *Z* = 2.52, *p*_bonferroni_ = 0.036.

**Table 4.** Mean Scores And Standard Deviations on the SIAQ Components, Per Category of the Nominal Variables.

	*n* (%)	Solidarity	Satisfaction	Centrality	Self-definition
Gender identity					
Man, mean (*SD*)	470 (32.57%)	4.94 (1.29)	3.79 (1.41)*,^a^	5.09 (1.21)	3.78 (1.13)
Woman, mean (*SD*)	783 (54.26%)	5.04 (1.21)	3.51 (1.34)*,^a^	5.11 (1.24)	3.58 (1.07)
Diverse, mean (*SD*)	190 (13.17%)	5.03 (1.33)	3.66 (1.36)	5.22 (1.19)	3.62 (1.06)
Intelligence level					
Low, mean (*SD*)	100 (6.93%)	4.88 (1.33)	3.65 (1.38)	5.08 (1.17)	3.83 (1.14)
Medium, mean (*SD*)	285 (19.75%)	4.97 (1.29)	3.41 (1.33)	5.16 (1.16)	3.73 (1.08)
High, mean (*SD*)	1006 (69.72)	5.03 (1.24)	3.67 (1.37)	5.11 (1.25)	3.62 (1.09)
Language preference					
Person-first, mean (*SD*)	395 (27.37%)	4.88 (1.26)	3.57 (1.29)	4.91 (1.25)*,^b^	3.61 (1.08)
Identity-first, mean (*SD*)	137 (9.49%)	5.07 (1.28)	3.96 (1.42)	5.29 (1.12)*	3.73 (1.13)
Other, mean (*SD*)	41 (2.84%)	4.78 (1.56)	3.72 (1.37)	4.63 (1.47)*	3.43 (1.08)
Urbanicity					
Urban, mean (*SD*)	678 (46.99%)	5.00 (1.25)	3.60 (1.33)	5.16 (1.23)	3.65 (1.11)
In between, mean (*SD*)	237 (16.42%)	5.08 (1.24)	3.81 (1.36)	5.16 (1.14)	3.80 (1.05)
Rural, mean (*SD*)	292 (20.94%)	4.92 (1.32)	3.64 (1.46)	5.00 (1.29)	3.51 (1.10)
Education level					
Low, mean (*SD*)	129 (8.94%)	4.91 (1.46)	3.79 (1.57)	5.12 (1.30)	3.69 (1.16)
Medium, mean (*SD*)	490 (33.96%)	4.97 (1.30)	3.63 (1.37)	5.16 (1.16)	3.69 (1.09)
High, mean (*SD*)	803 (55.56%)	5.05 (1.19)	3.59 (1.34)	5.10 (1.25)	3.62 (1.08)

Kruskal-Wallis test was used to test for group differences (*p*-value <0.0036 indicating significance). If significant, Dunn’s post-hoc test was used for which a Bonferroni correction was applied to correct for the number of categories that were compared. Values in bold with an asterisk indicate a significant difference.

a Scores for men were higher than for women.

b Scores for participants with an identity-first language preference were higher than those for participants with a preference for person-first or other.

Spearman’s correlations revealed significant associations of small strength with the SIAQ components with age, time since diagnosis, AQ score, anxiety, and depressive symptoms (see Table 5). Age was positively correlated with self-definition (*r_s_* = 0.08, *p* = 0.0026). Time since diagnosis was negatively related with solidarity (*r_s_* = −.09, *p* = 0.001) and centrality (*r_s_* = −0.13, *p* < 0.001). AQ scores were positively correlated with centrality (*r_s_* = 0.18, *p* < 0.001) and self-definition (*r_s_* = 0.18, *p* < 0.001). For anxiety and depressive symptoms, negative associations with satisfaction (anxiety: *r_s_* = −0.17, *p* < 0.001; depression: *r_s_* = −0.22, *p* < 0.001) were found, whereas positive associations with centrality were found (anxiety: *r_s_* = 0.16, *p* < 0.001; depression: *r_s_* = 0.09, *p* < 0.001).

**Table 5.** Spearman Rank Correlations with the SIAQ Components.

Variable	Solidarity	Satisfaction	Centrality	Self-definition
Age	<0.01	−0.02	<0.01	0.08*
Employment status^a^	−0.01	0.07	−0.05	−0.05
Ethnicity^b^	−0.01	<0.01	−0.03	−0.02
Age of diagnosis	0.03	−0.02	0.06	0.08
Time since diagnosis	−0.09*	0.03	−0.13*	−0.01
AQ scores	0.03	−0.05	0.18*	0.18*
Co-occurring conditions^c^	0.05	−0.06	0.06	<0.01
Anxiety symptoms	0.04	−0.17*	0.16*	0.06
Depressive symptoms	−0.04	−0.22*	0.09*	0.03

Significant correlations are presented in bold with an asterisk and refer to a *p*-value < 0.0036.

a Employment status was coded as 0 = *no paid job*, 1 = *paid job*.

b Ethnicity was coded as 0 = *non-Dutch* and 1 = *Dutch*.

c Co-occuring conditions were coded as 0 = *no*, 1 = *yes*.

The correlations among all variables are shown in Supplemental Table 5.

## Discussion

Understanding how autistic people relate to their classification and the autistic community, that is, their social identity, may offer new perspectives on mental health. As research has been limited by a lack of well-studied tools to measure social identity in autistic people, this study evaluated the SIAQ in the Netherlands. In addition, it was explored how social identity relates to demographics, autism characteristics, and mental health. Our findings, based on a large sample of autistic individuals, demonstrate that the SIAQ is a robust tool to assess social identity in people with autism in the Netherlands, thereby capturing four factors: *solidarity* (feelings of connection to people with autism), *satisfaction* (positive feelings about being autistic), *centrality* (the importance of autism to one’s sense of self), and *self-definition* (perceived similarity to and within the autistic community), which differentially relate to demographics, autism characteristics, and mental health.

### Psychometric Evaluation

The factor analysis showed that all 14 items clearly belonged to one of the four components, with high primary loadings and no substantial cross-loadings. Internal consistency was acceptable to excellent across all components, indicating reliable measurement of each construct. Measurement invariance testing demonstrated that the SIAQ performs equivalently across age, gender, education level, ethnicity, and autism traits. This indicates that people from different backgrounds interpret the items in similar ways, allowing for meaningful comparisons of subscale scores between groups. However, it is important to note that this study was carried out in the Netherlands and that cultural differences between countries could influence how autistic social identity is experienced and how items are interpreted, highlighting the need for cross-cultural validation in future research. Such measurement equivalence is particularly valuable given the wide variety of individuals with autism. Together, these findings provide strong support for the SIAQ as a valid instrument that can be used in clinical assessment and research applications.

### Theoretical Alignment

Our four-factor structure aligns with the initial theory-driven model proposed by Leach and colleagues (2008). They used the same questionnaire to assess various social identities (e.g. ethnicity, being a student) in the Netherlands and identified five subcomponents, grouped within two broader dimensions. At first glance, their factor structure appeared different from ours. However, on closer inspection, important similarities arise. Their first dimension, ‘self-investment’, includes solidarity, satisfaction, and centrality – which correspond directly to the first three components identified in our analyses. Their second dimension, ‘self-definition’, is identical to the fourth component that we identified. While Leach and colleagues (2008) identified two subcomponents within self-definition, we did not. This difference may be explained by methodological choices: Leach et al. (2008) tested hierarchical models, while we chose not to. Our primary objective was to establish clear guidelines for questionnaire implementation in both research and clinical practice – that is, to determine how items should be aggregated into meaningful subscales. Although hierarchical models can provide valuable theoretical insights, they may complicate practical applications by creating ambiguity around subscale construction. Our nonhierarchical approach facilitates more straightforward interpretation and subscale creation, thereby enhancing the instrument’s utility for both research and clinical practice. Based on our findings, we recommend using four subscales to assess social identity in autism.

### Autism as a Social Identity

The current findings demonstrate that autistic social identity follows the same structural patterns as other social identities (Leach et al., 2008), suggesting that autism can be meaningfully understood as a social identity, and not only as a neurodevelopmental condition. This alignment opens the door to applying established social identity theories to autism, offering new directions for research and a framework for understanding how social identity in autism and mental health are related. The Social Identity Theory (Tajfel & Turner, 1979) explains that individuals derive their self-concept not only from personal achievements but also from their group memberships. This suggests that societal portrayals of the autistic community can influence individual wellbeing, even for those who do not personally experience discrimination. Deficit-focused views and negative media representations can thus impact mental health of the autistic community, underscoring the importance of strength-based approaches that frame autism as neurodiversity rather than pathology. Promoting wellbeing in autistic individuals therefore requires not only individual interventions but also societal attention to how autism is portrayed and understood. It is important to note that societal portrayals of autism differ per country and culture, and that a culturally sensitive approach to promote wellbeing is crucial.

### Social Identity and Demographics

Analyses revealed significant associations of small strength between social identity and individual characteristics. Participants who identified as man reported higher satisfaction than self-identified women. This indicates that self-identified men viewed their autism more positively – perceiving it as something pleasant and worthy of pride – than self-identified women. For women, cultural and gendered expectations that position women as particularly sociable (Yau et al., 2023) may pressure women to mask their autistic traits (i.e. make eye-contact) to avoid being perceived as unsociable. Although masking is described by autistic people as necessary to survive in a neurotypical world, it is not without costs: masking has been associated with increased mental health problems (Bradley et al., 2021), and autistic women report more masking and more mental health issues than autistic men (Alaghband-rad et al., 2023; Martini et al., 2022; McQuaid et al., 2022). Masking may also disrupt identity formation; women with autism describe that masking leads to confusion about their identity and sense of self (Yau et al., 2023). This underscores the need for supporting positive identity formation in autistic women, recognizing their unique challenges and strengths.

Older age was related to higher self-definition, suggesting that older participants perceive greater similarity among people with autism and feel more similar to other autistic individuals than younger participants. This is in line with prior findings that adults are more likely to have developed an autistic identity and to have searched for support in the autism community (Gray et al., 2024). It may therefore be that an older age increases the likelihood of defining oneself as part of the autism community and to consider people with autism as one group, which is reflected by higher scores on self-definition. However, older participants did not necessarily receive a diagnosis earlier in life, as many autistic people are diagnosed later. To explore this further, we also studied time since diagnosis in relation to social identity.

### Social Identity and Autism Characteristics

Time since diagnosis, one of the autism-related variables, was not related to self-definition and satisfaction but was negatively related with solidarity and centrality. This indicates that participants diagnosed longer ago earlier felt less connected to other people with autism (solidarity) and considered autism as less central to their identity (centrality). This may be explained by the shift from the deficit-based approach toward the strength-based approach over the past decade (Murthi et al., 2023). Individuals that received their formal classification more recently may have had more exposure to a positive, strength-based view of autism. This might strengthen their connection to other people with autism (solidarity) as well as the significance of autism to one’s sense of self (centrality). Another explanation may be that the earlier longer ago someone received their autism classification, the less autism is at the forefront of one’s mind. Therefore, someone might think less about autism (centrality) and feel a less strong connection to the autism community (solidarity). More research is needed to understand the timing of diagnosis in relation to social identity, thereby also considering societal perceptions and the individual’s immediate environment (e.g. attitudes of family members and close friends toward autism).

Another autism-related variable that showed significant associations with social identity was language preference. People who preferred identity-first language (e.g. ‘autistic person’) reported higher centrality, which indicates that individuals who see autism as central to their identity use language that affirms that connection. This aligns with previous research showing that a stronger autism identity is associated with a stronger preference for identity-first language (Bury et al., 2022).

Furthermore, participants with more autism traits reported autism as more central to their identity (centrality) and endorsed greater similarity to and within people with autism (self-definition) than participants with less autism traits. Previous research indicated that stronger autism traits were related to stronger autism identification (Bury et al., 2022; Cooper et al., 2023). This could indicate that individuals who recognize more autistic traits in themselves may perceive autism as more central to their identity (centrality) and perceive people with autism as more similar to each other and to themselves (self-definition). Alternatively, a stronger identification with autism and other autistic people may encourage individuals to better recognize and acknowledge their autistic traits.

### Social Identity and Mental Health

With respect to mental health, higher anxiety and depression symptoms were associated with lower satisfaction and higher centrality. This aligns with Cooper et al. (2023) who found that higher centrality was associated with more social anxiety, and that higher satisfaction predicted lower social anxiety and higher wellbeing. The relationship between satisfaction and mental health seems quite straightforward given that a positive self-view is generally considered crucial to wellbeing (Sowislo & Orth, 2013). The findings regarding centrality may involve other underlying, moderating, and/or mediating factors. A potential underlying factor is stigma around autism, which could heighten autistic self-awareness (higher centrality) while simultaneously causing mental health problems (Botha & Frost, 2020). Masking may form a mediator: identifying more with the autistic community was related to more masking (Davies et al., 2024), which can increase mental health problems (Bradley et al., 2021). Furthermore, autistic individuals’ perspective on autism may be a moderator: Holding a deficit-based view may result in more mental health problems, while a strength-based view may buffer against mental health difficulties (Ferenc et al., 2023).

### Implications

The SIAQ offers researchers and clinicians a robust tool for assessing social identity in autism across diverse autistic individuals in terms of age, gender identity, ethnicity, education level, and autism traits. This is crucial for gaining a better understanding of how social identity is related to mental health. Our findings suggest that fostering positive aspects of autistic identity, such as satisfaction, may support mental health among autistic people in the Netherlands. Interventions such as peer support and identity-affirming education may help individuals develop a positive sense of self. This aligns with recent Australian research showing that newly diagnosed autistic adults find value in neurodiversity-affirming resources that validate their identity and offer a sense of community – while highlighting the need for more structured support and guidance (Edwards et al., 2025).

### Strengths, Limitations, and Future Research

A key strength of this study is the large and diverse sample. However, Dutch and highly intelligent and educated participants were overrepresented. Although measurement invariance across ethnicity and education was established – suggesting negligible bias in the SIAQ’s structure and functioning – it remains important to include more diverse participants in future research on social identity and wellbeing. Further research should explore which factors shape this relationship, such as time since diagnosis, stigma, masking, cultural background, and life stage. Longitudinal and qualitative research may be particularly valuable in understanding how autistic identity evolves and interacts with these contextual influences. Another potential limitation may be that the Dutch translation of the SIAQ included both person-first and identity-first language, meaning that some items did not fully align with individual language preferences. Although this could theoretically have influenced responses, our findings suggest otherwise: Centrality scores were higher among those preferring identity-first language even though most centrality items used person-first wording. Furthermore, items did not cluster by language. Instead, the close alignment with previous theory-driven models indicates that language preference did not bias responses (Leach et al., 2008). Furthermore, a potential limitation is the use of self-estimated IQ when IQ test scores were unavailable. However, previous research with NAR participants has found correlations of moderate strength between self-estimated and tested IQ scores in autistic individuals, which is considered sufficient for group-level analyses (Van der Burg et al., 2025). Given our large sample, using self-estimated IQ when test scores were unavailable was therefore considered reasonable and methodologically sound. Our findings underscore the importance of distinguishing between different aspects of social identity, as they show distinct associations with mental health (i.e. satisfaction linked to lower anxiety and depressive symptoms, but centrality to higher symptoms). Finally, to confirm the SIAQ as a robust instrument, further validation is needed, including test–retest reliability and convergent/divergent validity, using appropriate measures depending on the study’s design.

## Conclusion

In sum, this study shows that the SIAQ is a suitable tool for measuring social identity in autistic individuals in the Netherlands, offering insights into its association with mental health. The results show that the SIAQ forms a scientifically grounded method to advance research and practice that supports autistic identity and wellbeing, thereby aligning with priorities voiced by autistic people themselves (Cage et al., 2024; Edwards et al., 2025).

## Supplemental Material

sj-csv-1-aut-10.1177_13623613261431269 – Supplemental material for Assessing Social Identity in Autistic Individuals: Evaluating A Self-Report Questionnaire in the Netherlands Supplemental material, sj-csv-1-aut-10.1177_13623613261431269 for Assessing Social Identity in Autistic Individuals: Evaluating A Self-Report Questionnaire in the Netherlands by Lisa JG Krijnen, Ralph CA Rippe, Sander Begeer and Rachel D Plak in Autism

sj-docx-1-aut-10.1177_13623613261431269 – Supplemental material for Assessing Social Identity in Autistic Individuals: Evaluating A Self-Report Questionnaire in the Netherlands Supplemental material, sj-docx-1-aut-10.1177_13623613261431269 for Assessing Social Identity in Autistic Individuals: Evaluating A Self-Report Questionnaire in the Netherlands by Lisa JG Krijnen, Ralph CA Rippe, Sander Begeer and Rachel D Plak in Autism

## Notes

1. Both identity-first language (autistic person) and person-first language (person with autism) are used, since preferences vary among people with autism (Buijsman et al., 2023).
