# Supplementary material for: Assessing Social Identity in Autistic Individuals: Evaluating A Self-Report Questionnaire in the Netherlands
Source: Autism. 2026 Apr 3;30(5):1317–29. doi: 10.1177/13623613261431269 (PMC13087165; doi:10.1177/13623613261431269)
Supplement: sj-docx-1-aut-10.1177_13623613261431269 – Supplemental material for Assessing Social Identity in Autistic Individuals: Evaluating A Self-Report Questionnaire in the Netherlands [file sj-docx-1-aut-10.1177_13623613261431269.docx]

## Supplementary Materials

### **Statistical analysis details**

First, factor analyses were performed to determine the factor structure of the SIAQ. The sample was randomly split in 60%/40% to create a training sample (60%) to perform an exploratory factor analysis (EFA), and a validation sample (40%) to perform a confirmatory factor analysis (CFA).
 On the training sample, it was first checked whether the correlations among the items were suitable to perform an EFA. This was done by checking the strength of the correlations (too low if *r* <.30 with all other items and too high if *r* >.90 with another item), calculating the determinant (>.0001 indicates no multicollinearity), performing the Bartlett’s test (significant results indicates suitable strength and differentiation of correlations), and a Kaiser-Meyer-Olkin (KMO) test which provides a Measure of Sampling Adequacy (MSA) which measures the degree to which an item is predicted by other items without error (MSA >.50 indicates acceptable patterns of correlations among the items) (Field et al., 2012). Next, to determine the number of factors, the eigenvalues (Sum of Squares (SS) loadings >1.00 indicate a separate component according to Kaiser’s rule) were calculated and parallel analysis with 20 random sets were performed using ‘psych’ package (R). Hence, an EFA, using the ‘GPArotation’ package (R), was performed using an oblique rotation method (oblimin) as factors were theoretically expected to be correlated - i.e., all factors aim to measure (aspects of) identification with autism. Items with a factor loading of >.40 were considered to have a meaningful loading on a factor. Maximum Likelihood (ML) method of extraction was used.
 Next, a confirmatory factor analysis (CFA) was performed on the validation sample to confirm the results found in the EFA, using the ‘Lavaan’ package version 06-19 (R). Maximum Likelihood method of extraction was used and factors were allowed to correlated. Model fit was assessed using the following fit indices and thresholds for a good fit: Chi^2^ test (non-significant result), Tucker Lewis Index (TLI, >0.95), Comparative Fit Index (CFI; >0.95), Root Mean Square Error of Approximation (RMSEA; <0.08), Standardized Root Mean Squared Residual (SRMR; <.08) (Hu & Bentler, 1999). Although these thresholds are often used, they have been criticized as potentially overly strict (Perry et al., 2015) and therefore serve as guidelines rather than strict criteria.
 Internal consistency was calculated for both the training and validation sample using Cronbach’s alpha. Values were interpreted as follows: >0.70 acceptable, >0.80 good, >0.90 excellent (Taber, 2018).
 To test measurement invariance, the Lavaan package version 0.6-19 (R) was used. Using the total dataset, configural (equal factor structure), metric (equal loadings) and finally scalar (equal intercepts) invariance were tested across subgroups of gender (women, man, diverse), education level (low/medium/high), ethnicity (Dutch vs non-Dutch), age (> 1SD below the mean, between 1 SD below and above the mean, >1SD above the mean), and AQ-score (> 1SD below the mean, between 1 SD below and above the mean, >1SD above the mean). Due to gaps in response patterns on the SIAQ (e.g., no highly educated participants scored 7 on any of the SIAQ items), numerical estimators (MLR) were used and the data was treated continuous. Scaled fit indices (CFI, TLI, RMSEA, SRMR) were reported to correct for the ordinal nature of the data (i.e., Likert scale of 1-7). Standard fit indices have also been calculated to align with what the majority of previous research reports. Fit indices were interpreted as follows: CFI and TLI >.90, and preferably >0.95, RMSEA and SRMR <.08, and preferably <.05 (Ubels & Schlander, 2023; van de Schoot et al., 2012). To test whether the fit of the model was significantly worse compared to the previous model, differences in fit indices between models were tested. A not-significantly worse fit was concluded when at least 2 of the 3 criteria were met: CFI difference ≤0.01, SRMR difference ≤.01, RMSEA difference ≤ 0.015. In addition, the Scaled Chi-Squared Difference Test (method = “satorra.2000”) was performed to test whether the next model was not significantly worse than the previous model. However, this test is known for being easily significant in larger sample sizes (van de Schoot et al., 2012) and will therefore not be used as the primary decision criterion, but rather considered alongside the fit indices described above.
 Associations with the SIAQ components and other variables (demographic-, autism related- and mental health variables) were tested using non-parametric tests to protect against non-normality. For each SIAQ component, mean scores were calculated by summing the relevant items and dividing by the number of items. Mean scores were preferred over factor scores as the latter are sample-dependent, while mean scores facilitate direct comparisons with future research using the SIAQ. Spearman correlations were used for continuous and binary variables, while Kruskal-Wallis tests with Dunn's posthoc tests were conducted for categorical variables with at least 3 categories. Spearman’s correlations of <.30 were considered small, .30-.50 moderate and >.50 strong (Cohen, 1988). For the Kruskal Wallis test, *η*² was reported as a measure of effect size, with *η2* < 0.06 considered as small, .06-.14 medium and >.14 strong (Cohen, 1988). Correction for multiple testing was applied for the main tests (i.e., Kruskal Wallis and Spearman’s correlation) by adjusting the significance threshold: 0.05 was adjusted by the number of demographic-, autism-related-, and mental health variables tested (0.05/14 = .0036). For Dunn’s posthoc test, a Bonferroni correction was applied to correct for the number of categories that were compared.

### **Supplemental Table 1**. Interfactor correlations after oblimin rotation of EFA (lower diagonal) and of CFA (upper diagonal).

| **Factor** | Solidarity | Satisfaction | Centrality | Self-Definition |
| --- | --- | --- | --- | --- |
| Solidarity | .97 | .36 | .36 | .47 |
| Satisfaction | .26 | .99 | .24 | .23 |
| Centrality | .42 | .20 | .96 | .41 |
| Self-Definition | .43 | .22 | .43 | .97 |

### **Supplemental Table 2.** Measurement invariance

| **Gender^a^** | Chi^2^ | Df | p | CFI | Δ CFI | TLI | Δ TLI | RMSEA | ΔRMSEA | SRMR | Δ SRMR | Decision |
| --- | --- | --- | --- | --- | --- | --- | --- | --- | --- | --- | --- | --- |
| Model 1: Configural invariance | 819.72 | 213 | <.001 | 0.93 | NA | 0.91 | NA | 0.077 | NA | 0.057 | NA | Achieved |
| Model 2: Metric invariance | 833.32 | 233 | <.001 | 0.93 | 0.001 | 0.92 | 0.008 | 0.073 | -0.004 | 0.059 | 0.002 | Achieved |
| Model 3 Scalar invariance | 894.55 | 253 | <.001 | 0.93 | -0.005 | 0.92 | 0.001 | 0.073 | -0.001 | 0.060 | 0.001 | Achieved |
| **Education level^b^** |  |  |  |  |  |  |  |  |  |  |  |  |
| Model 1: Configural invariance | 814.03 | 213 | <.001 | 0.93 | NA | 0.91 | NA | 0.077 | NA | 0.058 | NA | Achieved |
| Model 2: Metric invariance | 837.28 | 233 | <.001 | 0.93 | 0.000 | 0.92 | 0.007 | 0.074 | -0.003 | 0.060 | 0.002 | Achieved |
| Model 3 Scalar invariance | 864.02 | 253 | <.001 | 0.93 | -0.001 | 0.93 | 0.006 | 0.071 | -0.003 | 0.061 | 0.000 | Achieved |
| **Ethnicity^c^** |  |  |  |  |  |  |  |  |  |  |  |  |
| Model 1: Configural invariance | 751.76 | 142 | <.001 | 0.93 | NA | 0.91 | NA | 0.084 | NA | 0.056 | NA | Achieved |
| Model 2: Metric invariance | 752.33 | 152 | <.001 | 0.93 | 0.001 | 0.91 | 0.007 | 0.081 | -0.003 | 0.057 | 0.001 | Achieved |
| Model 3 Scalar invariance | 763.32 | 162 | <.001 | 0.93 | 0.000 | 0.92 | 0.005 | 0.078 | -0.002 | 0.057 | 0.000 | Achieved |
| **Age^d^** |  |  |  |  |  |  |  |  |  |  |  |  |
| Model 1: Configural invariance | 845.81 | 213 | <.001 | 0.93 | NA | 0.91 | NA | 0.079 | NA | 0.058 | NA | Achieved |
| Model 2: Metric invariance | 872.22 | 233 | <.001 | 0.93 | -0.001 | 0.92 | 0.007 | 0.076 | -0.003 | 0.061 | 0.003 | Achieved |
| Model 3 Scalar invariance | 936.41 | 253 | <.001 | 0.92 | -0.005 | 0.92 | 0.001 | 0.075 | -0.001 | 0.062 | 0.001 | Achieved |
| **AQ-Score^e^** |  |  |  |  |  |  |  |  |  |  |  |  |
| Model 1: Configural invariance | 846.76 | 213 | <.001 | 0.93 | NA | 0.91 | NA | 0.079 | NA | 0.057 | NA | Achieved |
| Model 2: Metric invariance | 868.01 | 233 | <.001 | 0.93 | 0.000 | 0.92 | 0.008 | 0.075 | -0.003 | 0.060 | 0.001 | Achieved |
| Model 3 Scalar invariance | 898.08 | 253 | <.001 | 0.93 | -0.001 | 0.92 | 0.005 | 0.073 | -0.002 | 0.061 | 0.001 | Achieved |

Note. Scaled fit indices were reported, with the Bentler version for SRMR. Configural invariance was evaluated using absolute fit criteria (CFI/TLI ≥ 0.90, RMSEA/SRMR ≤ 0.08), while metric and scalar invariance required both absolute fit criteria and at least 2 of 3 relative change criteria: ΔCFI ≤ 0.01, SRMR ≤ 0.01, ΔRMSEA ≤ 0.015
^a^n = 1443; n group 1 (man) 470, n group 2 (woman) = 783, n group 3 (genderdiverse) = 190
^b^n = 1422, n group 1 (low education level) = 129, n group 2 (medium education level) = 490, n group 3 (high education level) = 803
^c^n = 1208, n group 1 (non-Dutch) = 36, n group 2 (Dutch) = 1172
^d^n = 1438; n group 1 (> 1 SD below the mean age) = 278, n group 2 (1 SD around the mean age) = 887, n group 3 (>1 SD above the mean age) = 273
^e^n = 1443; n group 1 (AQ score below -1SD) = 213, n group 2 (AQ score between –1SD and +1SD) = 1020, n group 3 (AQ score above +1SD) = 210

### **Supplemental Table 3.** Measurement invariance results using standard fit indices.

| **Gender^a^** | Chi^2^ | Df | p | CFI | Δ CFI | TLI | Δ TLI | RMSEA | ΔRMSEA | SRMR | Δ SRMR | Decision |
| --- | --- | --- | --- | --- | --- | --- | --- | --- | --- | --- | --- | --- |
| Model 1: Configural invariance | 921.94 | 213 | <.001 | 0.94 | NA | 0.92 | NA | 0.083 | NA | 0.057 | NA | Achieved |
| Model 2: Metric invariance | 944.30 | 233 | <.001 | 0.94 | 0.000 | 0.93 | 0.007 | 0.080 | -0.004 | 0.059 | 0.002 | Achieved |
| Model 3 Scalar invariance | 1004.13 | 253 | <.001 | 0.93 | -0.004 | 0.93 | 0.002 | 0.079 | -0.001 | 0.060 | 0.001 | Achieved |
| **Education level^b^** |  |  |  |  |  |  |  |  |  |  |  |  |
| Model 1: Configural invariance | 901.98 | 213 | <.001 | 0.94 | NA | 0.92 | NA | 0.083 | NA | 0.058 | NA | Achieved |
| Model 2: Metric invariance | 935.95 | 233 | <.001 | 0.94 | -0.001 | 0.93 | 0.005 | 0.080 | -0.003 | 0.060 | 0.002 | Achieved |
| Model 3 Scalar invariance | 958.31 | 253 | <.001 | 0.94 | 0.000 | 0.93 | 0.006 | 0.077 | -0.003 | 0.061 | 0.000 | Achieved |
| **Ethnicity^c^** |  |  |  |  |  |  |  |  |  |  |  |  |
| Model 1: Configural invariance | 770.51 | 142 | <.001 | 0.94 | NA | 0.92 | NA | 0.086 | NA | 0.056 | NA | Achieved |
| Model 2: Metric invariance | 785.49 | 152 | <.001 | 0.94 | -0.001 | 0.92 | 0.005 | 0.083 | -0.003 | 0.057 | 0.001 | Achieved |
| Model 3 Scalar invariance | 792.58 | 162 | <.001 | 0.94 | 0.000 | 0.93 | 0.005 | 0.080 | -0.003 | 0.057 | 0.000 | Achieved |
| **Age^d^** |  |  |  |  |  |  |  |  |  |  |  |  |
| Model 1: Configural invariance | 941.24 | 213 | <.001 | 0.94 | NA | 0.92 | NA | 0.084 | NA | 0.058 | NA | Achieved |
| Model 2: Metric invariance | 973.03 | 233 | <.001 | 0.93 | -0.001 | 0.92 | 0.006 | 0.081 | -0.003 | 0.061 | 0.003 | Achieved |
| Model 3 Scalar invariance | 1035.97 | 253 | <.001 | 0.93 | -0.004 | 0.93 | 0.002 | 0.080 | -0.001 | 0.062 | 0.001 | Achieved |
| **AQ-Score^e^** |  |  |  |  |  |  |  |  |  |  |  |  |
| Model 1: Configural invariance | 945.98 | 213 | <.001 | 0.93 | NA | 0.92 | NA | 0.083 | NA | 0.057 | NA | Achieved |
| Model 2: Metric invariance | 967.76 | 233 | <.001 | 0.93 | 0.000 | 0.92 | 0.007 | 0.081 | -0.004 | 0.060 | 0.001 | Achieved |
| Model 3 Scalar invariance | 993.34 | 253 | <.001 | 0.93 | 0.000 | 0.93 | 0.006 | 0.078 | -0.003 | 0.061 | 0.001 | Achieved |

Note. Standard fit indices were reported. Configural invariance was evaluated using absolute fit criteria (CFI/TLI ≥ 0.90, RMSEA/SRMR ≤ 0.08), while metric and scalar invariance required both absolute fit criteria and at least 2 of 3 relative change criteria: ΔCFI ≤ 0.01, SRMR ≤ 0.01, ΔRMSEA ≤ 0.015
^a^N = 1443; n group 1 (man) = 470, n group 2 (woman) = 783, n group 3 (genderdiverse) = 190
^b^n = 1422, n group 1 (low education level) = 129, n group 2 (medium education level) = 490, n group 3 (high education level) = 803
^c^n = 1208, n group 1 (non-Dutch) = 36, n group 2 (Dutch) = 1172
^d^n = 1438; n group 1 (> 1 SD below the mean age) = 278, n group 2 (1 SD around the mean age) = 887, n group 3 (>1 SD above the mean age) = 273
^e^n = 1443; n group 1 (AQ score below -1SD) = 213, n group 2 (AQ score between –1SD and +1SD) = 1020, n group 3 (AQ score above +1SD) = 210

### **Supplemental Table 4.** Mean scores and standard deviations per item.

|  |  | Item | Mean (SD) |
| --- | --- | --- | --- |
| Solidarity | 1 | I feel a bond with other people with autism | 5.04 (1.37) |
|  | 2 | I feel solidarity with the autistic community | 5.05 (1.41) |
|  | 3 | I feel committed to people with autism | 4.93 (1.38) |
| Satisfaction | 4 | I am glad to be autistic | 3.49 (1.67) |
|  | 5 | I think that people with autism have a lot to be proud of | 4.79 (1.47) |
|  | 6 | It is pleasant to be a person with autism | 3.13 (1.57) |
|  | 7 | Being autistic gives me a good feeling | 3.07 (1.58) |
| Centrality | 8 | I often think about the fact that I am a person with autism | 4.95 (1.55) |
|  | 9 | The fact that I am autistic is an important part of my identity | 5.38 (1.42) |
|  | 10 | Being a person with autism is an important part of how I see myself | 5.02 (1.50) |
| Self-definition | 11 | I have a lot in common with the average autistic person | 4.00 (1.36) |
|  | 12 | I am similar to the average person with autism | 3.45 (1.35) |
|  | 13 | People with autism have a lot in common with each other | 4.01 (1.35) |
|  | 14 | Autistic people are very similar to each other | 3.15 (1.35) |

### **Supplemental Table 5.** Correlation matrix among all variables.

See supplementary excel sheet.

### **References**

Centraal Bureau voor de Statistiek. (2021). *Standaard Onderwijsindeling 2021*. https://www.cbs.nl/nl-nl/onze-diensten/methoden/classificaties/onderwijs-en-beroepen/standaard-onderwijsindeling--soi--/standaard-onderwijsindeling-2021

Centraal Bureau voor de Statistiek (CBS). (2024). *Inwoners per gemeente*. https://www.cbs.nl/nl-nl/visualisaties/dashboard-bevolking/regionaal/inwoners

Cohen, J. (1988). *Statistical power analysis for the behavioral sciences* (2nd ed.). Hillsdale.

Field, A., Miles, J., Field, Z., & Angeles, L. (2012). *DISCOVERING STATISTICS USING R*. SAGE Publications.

Hoekstra, R. A., Vinkhuyzen, A. A. E., Wheelwright, S., Bartels, M., Boomsma, D. I., Baron-Cohen, S., Posthuma, D., & Van Der Sluis, S. (2011). The construction and validation of an abridged version of the autism-spectrum quotient (AQ-short). *Journal of Autism and Developmental Disorders*, *41*(5). https://doi.org/10.1007/s10803-010-1073-0

Hu, L., & Bentler, P. (1999). Cutoff criteria for fit indexes in covariance structure analysis: Conventional criteria versus new alternatives. *Structural Equation Modeling: A Multidisciplinary Journal*, *6*(1), 1–55. https://doi.org/10.1080/10705519909540118

Perry, J. L., Nicholls, A. R., Clough, P. J., & Crust, L. (2015). Assessing model fit: Caveats and recommendations for confirmatory factor analysis and exploratory structural equation modeling. *Measurement in Physical Education and Exercise Science*, *19*(1). https://doi.org/10.1080/1091367X.2014.952370

Taber, K. S. (2018). The Use of Cronbach’s Alpha When Developing and Reporting Research Instruments in Science Education. *Research in Science Education*, *48*(6), 1273–1296. https://doi.org/10.1007/S11165-016-9602-2

Ubels, J., & Schlander, M. (2023). Measurement invariance and adapted preferences: evidence for the ICECAP-A and WeRFree instruments. *Health and Quality of Life Outcomes*, *21*(1). https://doi.org/10.1186/s12955-023-02208-9

van de Schoot, R., Lugtig, P., & Hox, J. (2012). A checklist for testing measurement invariance. *European Journal of Developmental Psychology*, *9*(4), 486–492. https://doi.org/10.1080/17405629.2012.686740

Zigmond, A. S., & Snaith, R. P. (1983). TheHospital Anxiety and Depression Scale. *Acta Psychiatrica Scandinavica*, *67*, 361–370. www.bergop.info
